# Illinois State Dental Association

**Published:** 1868-07

**Authors:** H. J. Smith


### ILLINOIS STATE DENTAL ASSOCIATION.

The annual meeting of the Illinois State Dental Association was held in the Senate chamber of the State capitol commencing May 12, 1868.

At 11 o'clock A. M. the meeting was called to order by Dr. G. H. Cushing, President of the Association, Dr. M. S Dean acting as Secretary.

The roll was called and the following members answered to their names: M. S. Dean, Chicago; S. Babcock, Springfield; G. H. Cushing, Chicago; J. N. Crouse, Mt. Carroll; O. Wilson, Aurora; E. H. Kilbourne, Aurora; Geo. P. Kingsley, Freeport; A. W. French, Springfield; H. N. Lewis, Quincy, H. J. Smith, Quincy.

The proceedings of the last meeting were read and approved.

The following were elected members of the society:

T. D. Laughlin, Springfield; G. S. Miles, Jerseyville, G. V. Black, Jacksonville; Chas. Henry, Jacksonville; G. W. Rivers, Pittsfield; C. O. Dean, Quincy; F. E. Hansen, Winchester; N. C. Hunting, Lincoln; C. S. Smith, Springfield; C. K. Sawyer, Jacksonville; J. M. Hurt, Lincoln.

The society then adjourned until 3 o'clock, P. M.

AFTERNOON SESSION.

The society met at 3 o'clock, P. M. pursuant to adjournment, Dr. G. H. Cushing in the chair.

The election of officers for the ensuing year, resulted in the selection of the following:

*President*, E. H. Kilbourne, Aurora.

*Vice President*, G. P. Kingsley, Freeport.

*Secretary*, H. J. Smith, Quincy.

*Treasurer*, A. W. French, Springfield.

*Librarian*, M. S. Dean, Chicago.

*Executive Committee.*—H. N. Lewis, C. W. Rivers, C. O. Dean, J. N. Crouse, G. V. Black.

The newly elected President, Dr. E. H. Kilbourne, was introduced by the retiring President, who on taking the chair made a few appropriate remarks, thanking the society for the honor conferred upon him in selecting him to preside over their deliberations, and assuring the members that the society should receive his highest consideration and influence.

Dr. J. N. Crouse, the Treasurer, made his report which was received and adopted.

Dr. Cushing, chairman of the committee on the Association's seal, made a report which was adopted, and the committee discharged.

The reading of essays now being in order, the President, Dr. E. H. Kilbourne, offered a paper on the "Treatment of Six Year Molars," in which he attributed the early decay and loss of these teeth to defective nutrition, and the want of proper knowledge on the part of parents and guardians of the real character and value of these organs. The remedy for this latter evil lies within the power of the Dentist, whose important duty it is to instruct his patients in these matters upon all occasions. Regards the practicability of preserving these organs after the pulp has become exposed in children under 10 years of age by destroying and removing, extremely doubtful. Suggests the propriety of appointing a committee to prepare an article on this subject for popular reading to be published in the principal newspapers and public journals through the State, with the official endorsement of the society.

The society adjourned until 8 o'clock, P. M.

EVENING SESSION.

At 8 o'clock the society met as per adjournment. The President, Dr. Kilbourne in the chair.

On motion, the reading of the minutes of the previous session was suspended.

The chair announced the subject of the treatment of the "Six Year Molars" still under discussion. Papers were read by Drs. Swayne and Smith.

Dr. Swayne strongly urges the importance of the preservation of these teeth. Advocates the destruction of the pulp when exposed, by the arsenical paste—its thorough removal, and the subsequent filling of the cavity with cotton saturated with creosote and iodine.

Dr. Black believes the premature extraction of the six year molars results in the imperfect development of the jaw, with a corresponding loss of the facial expression. Regards the retention and preservation most necessary, if not permanently, at least until the age of maturity.

The subject was farther discussed by Drs. French, Crouse, Cushing and others.

The meeting adjourned on the arrival of a delegation from St. Louis, consisting of Drs. Forbes, Judd, Poore, McKellops, Eames, and Parks, until to-morrow at 10 o'clock, P. M.

### SECOND DAY—MORNING SESSION.

The society met pursuant to adjournment, President Kilbourne in the chair.

On motion of Dr. Miles the rules were suspended for the election of members. Dr. S. L. Edwards, of Griggsville, was proposed and elected a member.

On motion, Drs. H. J. McKellops and Poore, of St. Louis, were elected honorary members of this Association.

The subject under discussion was passed, and the next, "Plugging Pulp Cavities and Canals" announced by the chair.

Dr. M. S. Dean, read a paper on this subject in which he says: The nerves of the roots are supposed to be devitalized, and the roots and surrounding parts in a healthy condition. That this narrowed down the subject to preparing and filling these cavities. He considers this a simple operation when proper care is taken in preparing the cavities, as a rule. Condemns cotton, Hill's stopping, tin and wood, for this purpose. They are all destructible materials, and all penetrable by the fluids and gasses Cotton if saturated by creosote, would answer very well, until the creosote became dissipated, which it would certainly do sooner or later, and that other fluids would as certainly take its place, to decompose and generate gasses, destructive to the surrounding parts. Hill's stopping is a non-conductor, but he considered this of no practical importance, it might be used after the foramen had been sealed with gold. He thought that the entrance of the fluids into the canal by endosmotic force might be prevented somewhat by creosote and tannin, used in this treatment by entering the tubuli of the dentine, and fixing the albuminous matter which they may contain, rendering them impermeable to either fluids or gasses. His reasons for preferring gold to any other material, is because it is incorruptible, and non-irritant, easier carried to the apicial foramen, and if thoroughly packed shuts out the subtlest intruder.

Dr. C. S. Smith advocated the use of cotton and creosote for filling pulp cavities, and canals, and contended that the creosote forms with the animal matter of the tooth an insoluble compound thus closing the dental tubuli, and making the canal impervious to the secretion.

Dr. Black uses gold in the form of a ribbon rolled on a broach, and forces this into the canal to the apex of the root.

Remarks were made by Drs. Lewis, Cushing, Juda, McKellops, and others. Considerable diversity of opinion was expressed in regard to the materials used, and the manner of manipulating.

The meeting adjourned until 3 o'clock P. M.

SECOND DAY—AFTERNOON SESSION.

The society met at 3 o'clock, P. M., as per adjournment. President Kilbourne presiding.

The minutes of the morning session read and approved.

"Receding of the Gums in persons of middle age—Cause and Treatment" was announced by the chair as the subject next in order for discussion.

Dr. Wilson read a paper on this subject which elicited some remarks.

Dr. Judd thought our researches upon the subject had failed to discover a satisfactory cause of the disease. It was not known whether the gums receded by absorption, or are washed away by an acrid fluid secreted by the gum, and acting upon their structure; thought the use of a stiff tooth brush, with constitutional remedies would be the proper treatment in such cases.

Dr. Dean thought the absorption when not caused by local irritation was the result of defective nutrition.

Under suspension of the rules, Dr. Eames, of St. Louis, exhibited before the society the plaster models of a most interesting case, in which the nasal, palatal, vomer, and anterior portion of the superior maxillary bones were entirely destroyed and removed by Lupus. The disease was permanently cured by topical treatment.

Dr. Eames also exhibited a very ingenious apparatus that he is preparing to restore the lost parts, which promises to be a success.

Dr. Freeman offered a paper on the "Observed Effects of the Premature Extraction of Temporary Teeth." The subject was discussed at some length by Dr. Forbes, Cushing and others.

The society adjourned until 8 o'clock, P. M.

### SECOND DAY—EVENING SESSION.

The Association met at 8 o'clock, according to adjournment. Dr. E. H. Kilbourne, presiding.

The minutes of the morning session read and approved.

Dr. Crouse moved the election of delegates to the American Dental Association, be the first business in order for the morning session—carried.

"Anæsthesia" was announced by the chair the next subject in order.

Dr. Cushing read a paper upon the duties of the profession in the use of anæsthetics, in which he briefly reviewed the merits of the two principal agents now in use, viz: Chloroform and Nitrous-Oxide. As to their safety and desirability, citing chiefly from the experiments and deductions of Prof. Watt, of Cincinnati, and drawing from the evidence the conclusion that much greater caution was necessary in the preparation of Nitrous-Oxide than was generally used, and that its preparation and administration was undertaken by a vast number of incompetent and irresponsible parties; but, establishing upon such evidence as was attainable, the fact that Nitrous-Oxide is much safest as well as its preferable qualities over Chloroform in other respects, and concluded with an earnest appeal for a full investigation of the subject in order to secure simplicity and certainty in its preparation, to establish, if possible, a standard by which it may be easily tested, and by every safeguard that can be thrown around it, to place it upon the most assured ground of safety and reliability, under the conviction that eventually it would prove to be the greatest boon to suffering humanity which a merciful providence could vouchsafe.

Remarks were made on the merits of the different anæsthetic agents in use by Drs. Wilson, Judd, Freeman and others.

Adjourned until to-morrow at 10 o'clock, A. M.

### THIRD DAY—MORNING SESSION.

The society met pursuant to adjournment, at 10 o'clock, A. M. Dr. Kilbourne in the chair.

The reading of the minutes of the preceding meeting was, on motion, dispensed with.

The committee appointed to nominate delegates to the "American Dental Association" reported the following names: Drs. Chas. Henry, G. S. Miles, O. Ames, C. S. Smith, F. E. Hansen, H. J. Smith, S. L. Edwards, J. D. Kilbourne, O. Wilson, A. W. Freeman, J. N. Crouse, G. P. Kingsley, G. V. Black. The report was adopted, and the delegates empowered to fill all vacancies occurring in the delegation.

Dr. Black offered the following resolution which was adopted:

*Resolved*, That a committee of three be appointed to prepare an address to the people on the subject of the importance of the proper treatment of the "Six Year Molars," and that such address be presented to the next meeting of this body for their consideration.

On motion, Drs. Kilbourne, Black and Cushing were appointed said committee.

The following resolutions were offered by Dr. E. H. Kilbourne, President of the Association.

*Resolved*, That the Illinois State Dental Society hereby endorse, in full, the action of the American Dental Association, at its last session at Cincinnati, in relation to the report of the committee appointed by said Association to examine credentials of the delegates from the so-called St. Louis Dental College, which report justly declares, has no legal existence, and that a diploma from such institution would be utterly worthless.

*Resolved*, That this so-called Dental College so far from being an honorable institution of learning, is only a shop for manufacturing diplomas, which are hawked about as any other wares or merchandise are hawked about by hucksters, and that any one engaged in such nefarious practice is unfit for fellowship with honorable men.

*Resolved*, That as Dentists seeking the highest good of our noble profession, we will use all honorable means to build up our Dental Colleges that are laboring to advance the interests of Dental science.

The resolutions were unanimously adopted.

Dr. M. S. Dean, of Chicago, was selected to address the Convention at its next annual meeting.

The Association adjourned to meet at Quincy, Ill., on the second Tuesday in May, 1869.

H. J. Smith, D.D.S. Secretary.

